# Factors Affecting Unhappiness at School among Japanese Adolescents: An Epidemiological Study

**DOI:** 10.1371/journal.pone.0111844

**Published:** 2014-11-04

**Authors:** Hisayoshi Morioka, Osamu Itani, Yoshitaka Kaneita, Hajime Iwasa, Maki Ikeda, Ryuichiro Yamamoto, Yoneatsu Osaki, Hideyuki Kanda, Sachi Nakagome, Takashi Ohida

**Affiliations:** 1 Division of Public Health, Department of Social Medicine, Nihon University School of Medicine, Tokyo, Japan; 2 Department of Public Health and Epidemiology, Faculty of Medicine, Oita University, Oita, Japan; 3 Department of Public Health, Fukushima Medical University School of Medicine, Fukushima, Japan; 4 Division of Clinical Psychology, Health Care and Special Support, Graduate School of Education, Joetsu University of Education, Niigata, Japan; 5 Division of Environmental and Preventive Medicine, Department of Social Medicine, Faculty of Medicine, Tottori University, Tottori, Japan; 6 Department of Public Health, Shimane University Faculty of Medicine, Shimane, Japan; National Cardiovascular Center Hospital, Japan

## Abstract

**Background:**

Unhappiness at school is one of the main reasons for truancy among adolescents. In order to assess this problem more thoroughly in the context of Japanese adolescents, the present study examined the associations between feelings of unhappiness at school and lifestyle habits, school life realities, and mental health status.

**Method:**

This study was designed as a cross-sectional survey. A self-administered questionnaire was provided to students enrolled in randomly selected junior and senior high schools throughout Japan. We calculated the percentages of both junior and senior high school students who felt unhappy at school based on factors related to school life, lifestyle habits, and mental health status. Multiple logistic regression analyses were performed in order to examine the associations between those factors and students' feelings of unhappiness at school.

**Results:**

A total of 98,867 valid responses were analysed, 7.9% (Boys: 8.4%, Girls: 7.4%) of which came from students who responded that they felt unhappy at school. For both junior and senior high school students, the percentages of those who felt unhappy at school were significantly higher among those who had not yet decided on their future life course, who did not participate in extracurricular activities, did not eat breakfast every day, went to bed late, had used tobacco or alcohol in the previous 30 days, and had poor mental health compared with others. The results of multiple logistic regression analyses indicated that the adjusted odds ratios for feeling unhappy at school with regard to the above-mentioned factors were significantly high for both junior and senior high school students.

**Conclusions:**

The present results suggest that school employees and administrators must provide health guidance to students, considering that irregular lifestyle habits, lower school engagement, smoking, drinking alcohol, and poor mental health status are all associated with maladaptation to school among adolescents.

## Introduction

Truancy (i.e., skipping school without a valid excuse) is considered to be a serious problem among adolescents. In addition to lower academic performance [1]–[3], truancy is reportedly associated with various problems, such as alcohol, tobacco, or drug use [1], [4]–[8], mental illness [9], and delinquency and other types of crime [10]. It has also been reported that truant students earn less in their future lives than do non-truant students [11], are more likely to abuse substances such as alcohol [12], and are more prone to mental disorders, such as depression [12]. The percentages of truant junior high school students in Japan and the U.S.A. are reported to be 13% [13] and 6.2–35% [1], [2], [9], respectively, although the figures differ according to differences in the definition of truancy, the survey methods employed, and the lifestyle and economic status in individual countries. As truancy is a common problem among junior and senior high school students, establishment of preventive and corrective measures is required.

Indicators of potential adolescent truancy include feelings of maladaptation to school, such as unhappiness in the school environment and dislike of school. A government survey conducted on Japanese junior high school students indicated that 5.6% felt unhappy at school [14]. U.S.A. report indicated that approximately 30% of truant youths claimed that they disliked going to school [15]. In addition, a study of the reasons for school dropout in the U.S.A. indicated that 36.6% of high school students expressed a dislike for school [16]. Another U.S. study reported that students who stated that they liked school had lower risk of truancy than those who stated they did not [2]. Although truancy is a complex and heterogeneous issue [17], we think that unhappiness at school is one of the main reasons for truancy. Therefore, a scientific assessment of factors that might impact on school maladaptation would probably yield data that would be helpful for devising corrective measures to reduce such factors.

Feeling unhappy at school has also been reported to be associated with dangerous sexual behaviour [18]. Identifying various factors, including public health problems, that may affect adolescents' feelings of unhappiness at school might facilitate prompt corrective measures to prevent dissatisfaction with school by isolating public health problems. To our knowledge, few studies have reported the factors that are related to lifestyle habits, school life issues, and mental status in association with school maladaptation in adolescents, including unhappiness at school. In our study, we asked junior and senior high school students in Japan whether they felt happy at school; those who gave a negative response were defined as being unhappy at school. We then clarified the prevalence of unhappiness at school and its associated factors. This study is one of a series of nationwide surveys on lifestyle habits, such as drinking alcohol, smoking, eating, and sleep, in Japanese junior and senior high school students, and has been preceded by four other surveys conducted in 1996, 2000, 2004, and 2008 [19]–[22].

## Methods

### Sampling design and strategy

Two-stage a stratified sample with cluster selection method was employed. First, 10,785 junior high and 4,991 senior high schools (15,776 in total), as of May 2009, in Japan were registered for this study. Among them, 131 junior high (selection rate: 1.2%) and 113 senior high (selection rate: 2.3%) schools were randomly selected.

Probability-proportional-to-size sampling was employed so that the probability of selection was proportional to the number of enrolled students. The sample size was determined by referring to the response rates of schools and the ranges of confidence intervals (CIs) based on the variance of the results from the last four nationwide surveys of alcohol drinking and smoking behaviour.

In the Japanese education system, children enter primary school at the age of 6 years and leave after 6 years of study. They then enter junior high school for 3 years of study, followed by a further 3 years at senior high school. Primary and junior high school education is compulsory. In this report, the first to third years of junior high school are called the 7th to 9th grades, and the first to third years of senior high school are called the 10th and 12th grades.

### Procedure

We sent a letter requesting cooperation in our survey to the principal of each selected school, along with the same number of questionnaires and envelopes as that of students enrolled. At each school where the principal had approved participation in our survey, home room teachers delivered the questionnaires to the students. To protect the privacy of the respondents and to obtain frank responses, we requested the teachers to comply with the following guidelines: (1) not to make any positive or negative remarks on alcohol use before the survey, (2) not to go around the classroom peering at the questionnaire sheets over the students' shoulders while the students were filling out the questionnaires, and (3) not to open the envelopes, thus protecting students' privacy. Teachers were also requested to inform the students that they would not open the students' envelopes. To ensure compliance with these guidelines, implementation guides for the survey were delivered to the teachers. In addition, it was stated in the questionnaire that the completed questionnaires would not be seen by the teachers. After filling out the questionnaire, each student was requested to place the completed questionnaire in the envelope supplied and then seal it with an adhesive flap. The teachers were requested to send back the sealed envelopes to the Nihon University School of Medicine without opening them, in accordance with the guide. The survey was conducted between October 2010 and March 2011. The obtained data were accessible only from a PC managed in the Nihon University School of Medicine. In addition, access to personally identifiable data was limited to one person.

### Study subjects

The subjects of this study were 62,296 and 92,923 students enrolled in 131 and 113 randomly selected junior and senior high schools, respectively (total 155,219). Among the junior and senior high schools, 84 and 82 schools, respectively, returned responses (school cooperation rate: 64.1% and 72.6%, respectively). This means that a total of 166 out of 244 junior and senior high schools returned responses (overall school cooperation rate: 68.0%). Among 107,786 actual participants, 99,416 responded to the questionnaire (38,702 junior high school and 60,714 senior high school students). The proportions of students who responded to the questionnaire were 91.3% and 92.8% in junior and senior high schools, respectively, and 92.2% in total. The eventual response rates were 62.1% and 65.3% for junior and senior high schools, respectively, and 64.0% in total. These response rates were almost the same as those in a series of previous studies that we had conducted in the same manner. From the collected questionnaires, 549 were excluded because sex or grade was not specified or the responses were inconsistent.Data from the remaining 98,867 questionnaires (38,552 and 60,315 from junior and senior high schools) were analysed.

### Measures

The questions used in this study were developed on the basis of the questionnaires employed in the previous four surveys (1996, 2000, 2004, and 2008) [19]–[22]. With regard to school life, the following two questions were added to the questionnaire:

1. “Do you feel happy at school?”
2. “What is your plan for your future life course?”
3. “Do you participate in extracurricular activities?”

For Question 1, the following 3 options were provided: “Yes, I do”, “Yes and no”, and “No, I don't”. For Question 2, the following 7 options were provided: “high school”, “vocational school”, “college”, “university”, “postgraduate school”, “taking a job after leaving the current school”, and “not decided yet”. Those who selected “university” or “postgraduate school” were grouped as students who intended to go to university, and those who selected “high school”, “vocational school”, “college”, or “taking a job after leaving the current school” were grouped as students who did not intend to go to university. Thus, the participants were classified into 3 categories: “With and without intention to go to university” and “not decided yet”. For Question 3, the following 2 options were provided: “Yes, I do” and “No, I don't”.

With regard to tobacco and alcohol use, the following two questions were added to the questionnaire:

1. “How many days, in total, did you smoke in the past 30 days?”
2. “How many days, in total, did you drink alcoholic beverages in the past 30 days?”

For Questions 4 and 5, the following 7 options were provided: none, 1 or 2 days, 3–5 days, 6–9 days, 10–19 days, 20–29 days, and every day. For statistical analysis, students who selected “none” were classified into the “no” category, and those who selected one or more days as a response were classified into the “yes” category.

In addition, questions on personal information and lifestyle habits were added to the questionnaire to examine the associations between those factors and students' unhappiness at school. Specifically, questions pertaining to sex (boy/girl), grade in school (7th/8th/9th or 10th/11th/12th), eating breakfast (almost every day/sometimes/seldom), and bedtime (before or at/after midnight) were included.

The Japanese version of the 12-item General Health Questionnaire (GHQ-12) had been used to evaluate mental health status in the previous nationwide studies. The GHQ-12 is a self-administered questionnaire designed as a tool for screening mental diseases [23], [24]. It consists of 2 factors – “depression and anxiety” and “decrease in positive feeling” – and a total of 12 items (6 items for each factor). Four answer options are provided for each item; selection of the 2 options representing absence of a corresponding symptom yields a score of 0 points, and selection of the other 2 options (representing presence of a corresponding symptom) yields a score of 1 point. The total possible score on this scale ranges from 0 to 12 points. Higher total scores indicate poorer mental health. The GHQ-12 was initially developed for adults, but was consequently verified for use in adolescents [25], [26]. Some studies set the cut-off value for the GHQ-12 to 4 and considered a subject having 4 points or higher as having poor mental health [25]–[27]. Using the results of previous studies, one study reported that when one question was extracted from each of the two factors and the sum of their scores was calculated, their sensitivity and specificity was high (87.0% and 85.1%, respectively) when a cut-off point of 1 was regarded as indicative of poor mental health [28]. These methods and cut-offs were employed in another large-scale epidemiological study [22]. Based on the above facts and considering the simplicity of filling out a questionnaire, the following two questions were also included: “Have you felt more unhappy and depressed than usual in the past 30 days?” and “Have you been able to enjoy your normal daily activities more than usual?” A score of 0 was regarded as indicative of good mental health and 1 or higher was regarded as indicative of poor mental health.

### Data analysis

First, the percentages of students who felt happy or unhappy at school and those with a neutral opinion were calculated based on their sex and their grade in school. Next, the percentages of students who felt happy or unhappy at school and those with a neutral opinion were calculated with regard to each surveyed factor based on sex, and the calculations were made separately for both junior and senior high school students. Finally, using multiple logistic regression analysis, the adjusted odds ratio of each factor and its 95% confidence interval (95% CI) were calculated with regard to feeling unhappy at school, and the analysis was conducted separately for both junior and senior high school students. All analyses were performed using SPSS 16.0 for Windows.

### Ethics Statement

In the Ethical Guidelines for Epidemiological Studies jointly announced by the Ministry of Health, Labour and Welfare and the Ministry of Education, Culture, Sports, Science and Technology of Japan, personal information is defined as follows: information of a living individual, and the name, birthday, and other descriptions included in that information can be used to identify a specific individual. The present study used anonymous questionnaires to avoid identification of individuals and safeguard the privacy of the subjects. In addition, as the guidelines do not include provisions on how to treat minors, parental consent is not necessarily required in Japan. In this study, informed consent was obtained from each student participants, class teachers, and principals of school by written. The Ethics Committee of Nihon University School of Medicine specifically approved school/teacher consent (in lieu of parental consent) for minor participation in this survey. It was not mandatory for students at participating schools to respond.

## Results

### Descriptive profile of subjects

The basic attributes of the 98,867 valid responders are shown in Table 1. Students at junior and senior high schools who wished to go to university accounted for 14.1% and 56.1%, respectively, and 79.3% and 62.8% participated in extracurricular activities. Students at junior and senior high schools who ate breakfast every day accounted for 88.1% and 81.7%, respectively. Among the students overall, 68.3% and 42.2% at junior and senior high schools, respectively, went to bed at or before midnight. Junior and senior high school students who had smoked in the past 30 days accounted for 2.0% and 5.3%, respectively, and 9.0% and 17.7% had consumed alcohol in the past 30 days. Students at junior and senior high schools with poor mental health status accounted for 37.6% and 50.4%, respectively.

**Table 1:** Characteristics of the analyzed subjects.

	Junior high school		High school	
	N	%	N	%
**SEX**				
Boys	19,153	49.7	29,641	49.1
Girls	19,399	50.3	30,674	50.9
**Grade**				
Grade 7 and 10	13,041	33.8	21,444	35.6
Grade 8 and 11	12,816	33.2	20,168	33.4
Grade 9 and 12	12,476	32.4	18,466	30.6
Unknown	219	0.6	237	0.4
**Feeling happy/unhappy at school**				
Happy at school	25,547	66.3	36,976	61.3
Neither happy nor unhappy at school	9,930	25.8	17,191	28.5
Unhappy at school	2,514	6.5	5,174	8.6
Unknown	561	1.5	974	1.6
**Intending to study at university**				
Yes	5,446	14.1	33,845	56.1
No	26,064	66.6	20,358	33.8
Not yet decided	6,403	16.6	5,165	8.6
Unknown	639	1.7	947	1.6
**Participating in extracurricular activities**				
Yes	30,554	79.3	37,842	62.8
No	7,107	18.4	21,422	35.5
Unknown	891	2.3	1,051	1.7
**Having breakfast**				
Everyday	33,975	88.1	49,298	81.7
Sometimes	2,720	7.1	6,039	10.0
Seldom	1,386	3.6	4,136	6.9
Unknown	471	1.2	842	1.4
**Bedtimes**				
Before or at midnight	26,348	68.3	25,457	42.2
After midnight	11,532	29.9	33,876	56.2
Unknown	672	1.8	982	1.6
**Smoking (past 30 days)**				
No	37,729	97.9	56,994	94.5
Yes	756	2.0	3,178	5.3
Unknown	67	0.1	1,439	0.2
**Drinking (past 30 days)**				
No	34,685	90.0	49,353	81.8
Yes	3,484	9.0	10,643	17.7
Unknown	383	1.0	319	0.5
**Mental health status**				
Good	23,448	60.8	28,936	48.0
Poor	14,509	37.6	30,407	50.4
Unknown	595	1.6	972	1.6

### Prevalence of unhappiness by gender and grade in school

The percentages of students who felt happy/unhappy based on sex and grade in school are shown in Table 2. For all school grades put together, the percentages who felt unhappy based on all grades were 8.4% for boys and 7.4% for girls, that for boys being significantly higher (p<0.01). With regard to grade in school, the percentages of pupils who felt unhappy at school were highest (p<0.01) among boys in the 12th grade (9.9%) and among girls in the 10th and 11th grades (8.1%).

**Table 2:** Percentages of students who felt happy/unhappy at school, based on sex and grade in school.

		Feeling happy/unhappy at school					
		Happy at school		Neither/nor		Unhappy at school	
		N	%	N	%	N	%
**Boys** b	**Grade 7** a	4,298	68.5	1,620	25.8	361	5.7
	**Grade 8** a	4,079	64.8	1,748	27.8	472	7.5
	**Grade 9** a	4,155	68.2	1,511	24.8	430	7.1
	**Grade 10** a	6,139	59.8	3,184	31.0	950	9.2
	**Grade 11** a	5,842	59.9	3,021	31.0	889	9.1
	**Grade 12** a	5,412	61.0	2,581	29.1	880	9.9
**Girls** b	**Grade 7** a	4,512	69.1	1,680	25.7	338	5.2
	**Grade 8** a	4,154	65.3	1,738	27.3	469	7.4
	**Grade 9** a	4,214	67.8	1,571	25.3	429	6.9
	**Grade 10** a	6,941	64.1	2,999	27.7	881	8.1
	**Grade 11** a	6,380	63.1	2,907	28.8	819	8.1
	**Grade 12** a	6,126	66.0	2,431	26.2	729	7.9

Subjects for whom data were missing were excluded from the analysis.

a P value was calculated by χ^2^-test, 3 (Feeling happy/unhappy at school; happy at school, Neither nor, unhappy at school) ×6(Grade; Grade 7, Grade 8, Grade9, Grade 10, Grade 11, Grade 12), P<0.01.

b P value was calculated by χ^2^-test, 3 (Feeling happy/unhappy at school; happy at school, Neither nor, unhappy at school) ×2(Sex; Boys, Girls), P<0.01.

### Associations between various factors and feeling unhappy at school

The associations between the various factors related to lifestyle habits and mental status and unhappiness at school are shown in Tables 3 and 4. For both junior and senior high school students, the percentage of those who felt unhappy at school was highest (p<0.01) among students who had not yet decided their future courses and among students who seldom ate breakfast. The percentage of those who felt unhappy at school was higher (p<0.01) among students who did not participate in extracurricular activities, whose bedtimes were later than midnight, who had used either tobacco or alcohol in the past 30 days, and who had a poor mental health status in comparison with the others.

**Table 3:** Associations between each factor and feeling happy/unhappy at junior high school.

				Feeling happy/unhappy at school		
				Happy at school	Neither/nor	Unhappy at school
			N	%	%	%
**Total**			37,991	67.2	26.1	6.6
**Boys**	* **Intending to study at university**	Yes	2,888	70.3	23.0	6.7
		No	12,232	69.4	24.9	5.7
		Not yet decided	3,565	56.7	32.9	10.4
	* **Participating in extracurricular activities**	Yes	15,498	69.6	24.7	5.7
		No	3,102	55.2	32.9	11.9
	* **Having breakfast**	Everyday	16,677	69.5	24.8	5.6
		Sometimes	1,334	50.7	36.7	12.6
		Seldom	762	42.8	36.1	21.1
	* **Bedtimes**	Before or at midnight	13,758	70.3	24.5	5.2
		After midnight	4,901	58.5	30.4	11.1
	* **Smoking (past 30 days)**	No	18,298	67.6	26.1	6.3
		Yes	457	47.7	26.3	26.0
	* **Drinking (past 30 days)**	No	16,963	68.0	25.9	6.1
		Yes	1,630	59.8	27.6	13.6
	* **Mental health status**	Good	12,948	75.2	22.1	2.7
		Poor	5,759	49.0	35.1	15.9
**Girls**	* **Intending to study at university**	Yes	2,545	72.6	22.4	5.0
		No	13,778	68.3	25.7	6.0
		Not yet decided	2,811	58.0	31.7	10.2
	* **Participating in extracurricular activities**	Yes	14,995	65.7	27.2	7.1
		No	3,975	58.6	30.5	10.9
	* **Having breakfast**	Everyday	17,214	69.4	25.0	5.6
		Sometimes	1,377	50.5	37.5	12.0
		Seldom	616	49.0	31.5	19.5
	* **Bedtimes**	Before or at midnight	12,526	70.5	24.7	4.8
		After midnight	6,600	61.4	28.9	9.7
	* **Smoking (past 30 days)**	No	18,917	67.8	26.0	6.2
		Yes	273	39.9	36.3	23.8
	* **Drinking (past 30 days)**	No	17,251	68.6	25.5	5.9
		Yes	1,776	56.8	30.9	12.4
	* **Mental health status**	Good	10,452	79.6	18.8	1.6
		Poor	8,705	52.8	34.9	12.3

Subjects for whom data were missing were excluded from the analysis.

*P value was calculated by χ2-test, each factors(Intending to study at university, Participating in extracurricular activities, Having breakfast, Bedtimes, breakfast, Bedtimes, Smoking, Drinking, Mental health status), P<0.01.

**Table 4:** Associations between each factor and feeling happy/unhappy at senior high school.

				Feeling happy/unhappy at school		
				Happy at school	Neither/nor	Unhappy at school
			N	%	%	%
**Total**			59,341	62.3	29.0	8.7
**Boys**	* **Intending to study at university**	Yes	17,173	63.5	28.7	7.8
		No	9,125	58.9	31.1	10.0
		Not yet decided	2,656	43.2	38.9	17.9
	* **Participating in extracurricular activities**	Yes	19,157	65.7	27.2	7.1
		No	9,775	49.3	36.7	14.0
	* **Having breakfast**	Everyday	23,362	63.1	29.0	7.9
		Sometimes	3,102	50.6	37.2	12.2
		Seldom	2,545	45.1	35.3	19.6
	* **Bedtimes**	Before or at midnight	13,073	64.2	28.4	7.4
		After midnight	15,861	56.9	32.0	11.1
	* **Smoking (past 30 days)**	No	26,940	61.2	30.1	8.6
		Yes	2,039	46.4	34.0	19.6
	* **Drinking (past 30 days)**	No	23,711	61.2	30.4	8.4
		Yes	5,153	55.3	30.5	14.2
	* **Mental health status**	Good	16,412	69.7	25.9	4.3
		Poor	12,523	47.4	36.3	16.0
**Girls**	* **Intending to study at university**	Yes	16,607	69.8	24.5	5.8
		No	11,167	60.6	30.2	9.2
		Not yet decided	2,499	45.3	36.7	18.0
	* **Participating in extracurricular activities**	Yes	18,614	69.9	24.6	5.5
		No	11,580	55.4	32.5	12.2
	* **Having breakfast**	Everyday	25,821	66.8	26.5	6.8
		Sometimes	2,920	52.2	34.7	13.2
		Seldom	1,580	47.8	32.7	19.6
	* **Bedtimes**	Before or at midnight	12,305	67.3	26.3	6.4
		After midnight	17,950	62.3	28.5	9.2
	* **Smoking (past 30 days)**	No	29,248	65.3	27.3	7.4
		Yes	1,042	38.3	35.6	26.1
	* **Drinking (past 30 days)**	No	24,895	66.1	26.8	7.0
		Yes	5,282	56.1	31.1	12.8
	* **Mental health status**	Good	12,465	77.9	19.7	2.3
		Poor	17,803	54.9	33.1	12.0

Subjects for whom data were missing were excluded from the analysis.

*P value was calculated by χ2-test, each factors(Intending to study at university, Participating in extracurricular activities, Having breakfast, Bedtimes, breakfast, Bedtimes, Smoking, Drinking, Mental health status), P<0.01.

### Multiple logistic regression analysis of the association between feeling unhappy at school and various factors

The adjusted odds ratio (AOR) and its 95% confidence interval (CI) for the association between feeling unhappy at school and each of the factors related to lifestyle habits and mental status are shown in Table 5. Among junior high school students, the AORs for those who seldom ate breakfast, who had smoked in the past 30 days, or who exhibited poor mental health were higher than 2.00. In particular, the AOR was highest for students who claimed that their mental health was poor (AOR: 6.75, CI: 6.08–7.50).

**Table 5:** Multiple logistic regression analysis of the association between feeling unhappy at school and each of the factors.

	Junior high school		Senior high school	
	Unhappy at school		Unhappy at school	
	AOR	95%CI	AOR	95%CI
Sex*				
Boys	1.00		1.00	
Girls	0.71	0.65–0.78	0.68	0.64– 0.72
Grade*				
7 and 10	1.00		1.00	
8 and 11	1.21	1.09–1.35	0.95	0.88–1.02
9 and 12	0.83	0.74–0.94	0.88	0.81–0.95
Intending to study at university*				
Yes	1.00		1.00	
No	0.98	0.86–1.11	1.36	1.27–1.45
Not yet decided	1.79	1.54–2.08	2.34	2.14–2.56
Participating in extracurricular activities*				
Yes	1.00		1.00	
No	1.77	1.59–1.97	1.87	1.75–1.99
Having breakfast*				
Everyday	1.00		1.00	
Sometimes	1.59	1.39–1.82	1.27	1.16–1.39
Seldom	2.27	1.93–2.67	1.94	1.77–2.13
Bedtimes*				
Before or at midnight	1.00		1.00	
After midnight	1.45	1.32–1.59	1.21	1.13–1.29
Smoking (past 30 days)*				
No	1.00		1.00	
Yes	2.35	1.90–2.90	1.6	1.43–1.79
Drinking (past 30 days)*				
No	1.00		1.00	
Yes	1.39	1.22–1.58	1.26	1.14–1.32
Mental health status*				
Good	1.00		1.00	
Poor	6.75	6.08–7.50	4.51	4.19–4.86

Abbreviations: AOR = adjusted odds ratio, CI = confidence interval.

Feeling unhappy at school: Students who selected “No, I don't” in response to the question asking whether the respondent felt happy at school (answer options: “Yes, I do”, “Yes and no”, and “No, I don't”).

Subjects for whom data were missing were excluded from the analysis.

Sex, Grade, Intending to study at university, Participating in extracurricular activities, Having breakfast, Bedtimes, Smoking, Drinking, and Mental health status were used as covariance values.

For calculation of the P values, a multivariate analysis model was used, *P<0.01.

Among the high school students, the AORs for those who had not yet decided their future course or those who exhibited poor mental health were higher than 2.00. In particular, the AOR for students who claimed that their mental health was poor was the highest (AOR: 4.51, CI: 4.19–4.86).

## Discussion

We believe that the present sample is representative of Japanese adolescents because (1) the participating schools were selected randomly from among junior and senior high schools nationwide and (2) approximately 100,000 responses were analysed. To our knowledge, the present study is the first large-scale epidemiological study to have investigated the prevalence of unhappiness at school among junior and senior high school students and the factors associated with such feelings.

In this study, the proportions of junior and senior high school students who claimed to feel unhappy at school were 6.5% and 8.6%, respectively (Table 2). According to a government survey conducted on approximately 1,000 Japanese junior high school students, the proportion of those who claimed to feel unhappy at school was 5.6% [14], which was similar to the present result. The same survey also indicated that the proportion of male junior high school students who were unhappy at school was higher than that of female junior high school students, corresponding with our study's result. Although no other studies offer data on sex-based comparisons with regard to unhappiness at school, it may be determined that girls are more adaptive to school than are boys. As the total number of students attending junior and senior high schools in Japan is almost seven million [29], these reported answers and statistical analysis suggest that as many as several hundred thousand students may feel unhappy at school.

In the present study, the proportions of students who claimed to feel unhappy at school were significantly higher among those who had not yet decided their future course, who did not eat breakfast every day, who had used tobacco or alcohol in the past 30 days, who did not participate in extracurricular activities, had late bedtimes, or who had poor mental health compared to the others (p<0.01; Tables 3 and 4). When dealing with students who fall into the above-mentioned categories, educators and administrators alike should consider that such students might not be able to adjust well to school. In addition, when teachers and parents develop measures to improve students' adaptivity to school, they must plan comprehensive measures, while also considering students belonging to the above-mentioned categories. With regard to the percentage of students who felt unhappy at school, the same factor trends were observed among both junior and senior high school students (Tables 3 and 4). The only factor that indicated a different trend between junior and senior high school students was an intention to study at university. The circumstances surrounding maladaptation may be the same for both junior and senior high school students.

The sex-based AORs for unhappiness at school were significantly lower (p<0.01) in both junior and senior high school girls (Table 5). When providing counselling and guidance, teachers should bear in mind that male students tend to develop feelings of unhappiness at school more easily than do female students. With regard to the AORs for feeling unhappy at school based on grade, the AORs for 9th and 12th graders were the lowest. This result was not affected by differences in future course after leaving the current school, because it was adjusted for intention to attend university. To clarify which factors were responsible for the lowest AORs among 9th and 12^th^ grade students, further research on characteristics peculiar to students in these grades is required.

Intention to attend university and participation in extracurricular activities were considered to be factors pertaining to school engagement. For junior high school students who had not yet decided their future course and for senior high school students who had either not yet decided on their future course or had decided not to attend university, the AORs for feeling unhappy at school were significantly higher than for junior and senior high school students who did intend to go to university (p<0.01; Table 5). This result may suggest the importance of helping students decide their future courses early, and of supporting high school students who do not intend to go to university. The AORs for unhappiness at school among junior and senior high school students who did not participate in extracurricular activities were significantly higher (p<0.01) than for those who did. In school life, in addition to class attendance, participation in extracurricular activities is vitally important (Table 5).

Eating breakfast and bedtime were also factors that impacted on the lifestyle habits of students. The AORs for feeling unhappy at school among students who did not eat breakfast every day or who did not go to bed before midnight were significantly higher (p<0.01) than for those who did (Table 5). To deal with students' feelings of school maladaptation, intervention by students' family members, as well as teachers and administrators is important.

The AOR for feeling unhappy at school among students who used tobacco or alcohol was significantly higher (p<0.01) than for those who did not (Table 5). Henry et al. [30]reported that students who expressed poor school bonding (not feeling happy at school, and so on) were much more likely to use alcohol, and this fits with the results of the present study. Henry et al. [8] also reported that truancy was a significant predictor of initiating alcohol and tobacco use. Generally, as feeling unhappy at school is a cause of truancy, the stress caused by school maladaptation may lead students to start using tobacco or alcohol. The need to improve the adaptivity of adolescents to school was suggested in order to prevent them from using tobacco or alcohol.

The AOR for feeling unhappy at school among students in poor mental health was significantly higher (p<0.01) than the AOR for those in good mental health (Table 5). This AOR was considerably higher than the AORs found for the other factors. It was confirmed that, in comparison with other factors related to lifestyle habits and school life, mental health was most strongly associated with unhappiness at school. Egger et al. [9] reported that truant adolescents were more likely to exhibit depression. One of the mental health questions in the present questionnaire was similar to the GHQ-12 question on depressive symptoms. As feeling unhappy at school may be associated with depression. As feeling unhappy at school may be associated with depression, teachers and parents may need to view such feelings as an important adolescent mental health issue and take appropriate measures to prevent it.

The following two suggestions can be provided based on the results of the multiple logistic regression analyses regarding adolescents' unhappiness at school. First, when providing healthcare guidance to students, school employees and administrators must consider that adolescents' irregular lifestyle habits, low school engagement, tobacco or alcohol use, and poor mental health are all associated with school maladaptation and possible future truancy. Second, family members of adolescents must be aware that irregular lifestyle habits, tobacco or alcohol use, and poor mental health are associated with school maladaptation. If it is suspected that a child feels unhappy at school because of insufficient nutritional intake or lack of sleep, his/her family is required to take corrective measures, such as helping their children lead regular lives.

The present study had some limitations. First, as it was cross-sectional in design, causal relationships could not be determined. For instance, a student may not have participated in extracurricular activities because he/she felt unhappy at school. On the other hand, a student may have felt unhappy at school because he/she did not participate in extracurricular activities. Longitudinal studies will be needed to elucidate the causality of this issue. Second, data from students who were absent from school on the survey day and school dropouts were excluded from the analyses. The percentage of students who felt unhappy at school may have been higher among absentees and dropouts than it was among those who were in attendance on the day of the study. The Japanese Education Ministry has reported that the proportions of junior and senior high school students who are absent from school 30 days or more per year for reasons other than illness or economic difficulties are 2.6% and 1.7%, respectively [31]. The dropout rate among high school students in this survey was 1.6% [31] (that among junior high school students is not available because junior high school education is compulsory in Japan). Thus, the proportions of students who felt unhappy at school may have been underestimated. Moreover, the data regarding the nine factors investigated in the present study may have been biased. Third, we only investigated the associations between feeling unhappy at school and each of nine factors. This study did not examine all of the elements that may have possibly affected feelings toward school and their relationships with each of the nine factors of interest. For instance, somatic complaints, including abdominal pain and headache, or externalized behaviour problems such as defiance and noncompliance were not assessed. Many more factors that could affect happiness at school must be included in future studies. Fourth, happiness is a subjective emotion. Different students may feel very differently about the same circumstances, depending on their own characteristics and customs. Therefore, the possibility of introducing an objective scale that can be used effectively in a large-scale epidemiological survey must be considered. Lastly, limited space on the questionnaire allowed us to pose only two questions from among 12 on the GHQ as a mental health measure. If more weight must be given to adolescents' mental health status in future surveys, all 12 GHQ questions may need to be included for more accurate measurement.

## Conclusions

This large-scale epidemiological study of junior and senior high school students in Japan has revealed that feeling unhappy at school is associated with being male, having an undecided future course, not participating in extracurricular activities, not eating breakfast every day, going to bed late, using tobacco or alcohol, and being in poor mental health. Policy makers must promote further investigations into how feeling unhappy at school is associated with the initiation of tobacco or alcohol use or the onset of mental disorders such as depression.
